# An antibacterial and biocompatible multilayer biomedical coating capable of healing damages†

**DOI:** 10.1039/d0ra04457a

**Published:** 2020-08-28

**Authors:** Yongxun Zhao, Yuan Liang, Qianqian Zou, Libin Ma, Yuping Wang, Yanxi Zhu

**Affiliations:** School of Life Sciences, Lanzhou University Lanzhou 730000 PR China yongxunzh@163.com; The Seventh Department of General Surgery, The First Hospital of Lanzhou University Lanzhou 730000 PR China; Department of Gastroenterology, The First Hospital of Lanzhou University Lanzhou 730000 PR China; Laboratory Department, Linyi City Hospital of Traditional Chinese Medicine Linyi Shandong 276000 PR China; Key Laboratory for Gastrointestinal Diseases of Gansu Province, Lanzhou University Lanzhou 730000 PR China; Central Laboratory of Linyi People's Hospital Linyi 276003 PR China zhu-yanxi@163.com

## Abstract

Besides the excellent biocompatibility and high antibacterial property, multifunctional biomedical coatings with a long service time is highly desirable for extended applications, which is still an ongoing challenge. The self-healing property enables new directions for effectively prolonging their service life and significantly improving their reliability. Herein, an efficient and simple method is used to facilely prepare antibacterial, biocompatibile multilayer polyelectrolyte coatings, which are capable of healing damages. The synthetic strategy involves the alternate deposition of Chitosan (CS) and sodium carboxymethyl cellulose (CMC) *via* the layer-by-layer (LBL) self-assembly technique. The CS/CMC multilayer polyelectrolyte coating features high antibacterial property, fast and efficient self-healing property, and excellent biocompatibility. These features allow the CS/CMC polyelectrolyte coating to have extended lifespan and to be highly promising for novel functional stent coating applications.

## Abstract 2

A CS/CMC multilayer polyelectrolyte coating was developed, which features fast and efficient self-healing property, high antibacterial property, and excellent biocompatibility.

## Graphical abstract

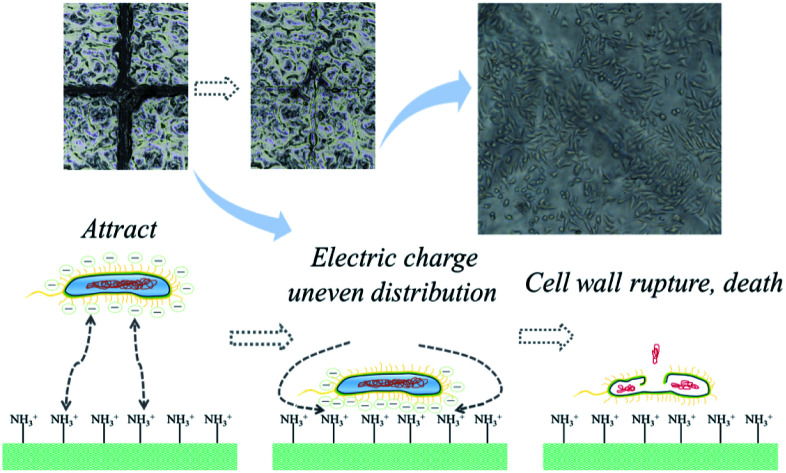

## 1 Introduction

Biomedical polyelectrolyte coatings are a type of polymers made from readily available biomaterials,^1^ which have good biocompatibility and hypoallergenic properties, and provide a sterile environment on their surface.^2^ These materials have been frequently applied in medical device protection,^3–5^ such as surgical suture coating, medical catheter coating, and surgical stent coating. Recently, there have been several reports on various types of polyelectrolyte stent coatings;^6,7^ however, their further application in clinical environments is greatly limited by the deterioration and accumulation of damages of the coatings during applications.^8,9^ Therefore, the designation and preparation of durable functional polyelectrolyte stent coatings are crucial.^10^ One of the most efficient strategies for the inhibition of damages of the polyelectrolyte stent coatings primarily involves on chemical polymerization and crosslinking.^11^ Although polymerization or the crosslinking technique can significantly improve the stability of the polyelectrolyte stent coatings;^12^ the special crosslinking agents in the composites have a certain poisonous side effect.^13^

Ideally, if the biomedical polyelectrolyte coatings are designed with self-healing property, similar to the ubiquitous organism in nature, which can heal injury and recover functionality spontaneously, the biomedical polyelectrolyte coatings can effectively prolong their service life and significantly improve their reliability.^14–17^ In the past decades, although different styles of self-healing polymeric multilayer coatings have been prepared,^18–20^ achieving multifunctional biomedical coatings with long service time is also highly desirable for its expansion applications, yet remains an ongoing challenge.

Herein, a highly efficient and technically simple approach is developed to synthesize antibacterial, biocompatible biomedical coating capable of healing damages. The synthetic strategy involves the alternate deposition of CS and CMC *via* the LBL self-assembly technique. As both the CS and CMC are derived from organisms, the composite materials had high biocompatibility. Moreover, CS and CMC are bonded by hydrogen bonds and electrostatic attractions, and these reversible non-covalent bonds can be broken and recombined under specific stimuli. These features make CS/CMC polyelectrolyte coatings to have extended lifespan and to be highly promising for applying in novel functional biomedical materials.

## 2 Experimental

### 2.1. Chemicals and materials

Sodium carboxymethyl cellulose (CMC, which viscosity is 300–800 mPa s), Chitosan (CS, *M*_w_ = 375 000, the degree of deacetylation of chitosan), acetic acid and sodium hydroxide were all purchased from Sinopharm Chemical Reagent Co., Ltd. The glass substrate was purchased from Sinopharm Chemical Reagent Co., Ltd. The CMC solution with the concentration of 4 mg mL^−1^ was prepared, and then 4 mg mL^−1^ of the CS solution, whose pH value ranged from 4 to 2.5, were prepared.

### 2.2. Fabrication the CS/CMC polyelectrolyte multilayer coating

The CS/CMC polyelectrolyte coatings were fabricated *via* the LBL assembly technique by Zhu *et al.*^21^ First, a glass substrate was immersed in the CS solution for 10 min to obtain the glass-CS, and then it was washed 5 times to take off the residue CS solution. Second, the glass-CS was immersed into the CMC solution for 10 min to obtain the glass-CS/CMC*1 polyelectrolyte coating, then it also was washed 5 times to wash off the residue CMC solution. Thirdly, the first and second process were repeated 39 times to obtain the glass-CS/CMC*40 polyelectrolyte multilayer coating. Finally, the CS/CMC*40 polyelectrolyte multilayer coating was peeled off from the glass substrate, and the CS/CMC polyelectrolyte multilayer coating was obtained.

### 2.3. Characterization

In this research, the FT-IR spectra of the CS, CMC and CS/CMC polyelectrolyte multilayer coating were analysed by Nicolet iS5 (Thermo Electron Scientific Instruments Corp). The wettability and surface topography of the CS/CMC polyelectrolyte multilayer coating were measured *via* inductively coupled plasma-atomic emission spectroscopy (ICP-AES, Shanghai Zhongchen Digital Technology Equipment Co., Ltd, Shanghai, China). The self-healing process of the CS/CMC polyelectrolyte multilayer coating was investigated *via* a Stereo Microscope (Olympus MVX10).

### 2.4. Antibacterial test

The CS/CMC polyelectrolyte multilayer coating was ground and milled to power, and 0.01 g was added to an agar solution to prepare LB Petri dishes, which was labelled as (b). Moreover, a normal LB Petri dish was used as the control sample, labelled as (a). The two LB Petri dishes were inoculated with the same content bacteria (*E. coli*), and then were cultured at 37 °C for 24 h.

### 2.5. Biocompatibility test

The CS/CMC polyelectrolyte multilayer coatings were cut into a circular membrane material with a diameter of 14 mm (the aperture of 24-well plate), and were closely attached to 24-well plate, marked as (b). Simultaneously, a normal 24-well plate was used as the control, marked as (a). After the samples were irradiated with UV for 30 min, L929 cells were added in them and cultured for 72 h, according to literature standards,^22^ and the growth of the L929 cells was observed by an inverted microscope.

In addition, the L929 cells were also cultured in 96-well plates according to the above procedure, and the CCK-8 test was performed according to the method in the literature.^23^

## 3 Results and discussion

**Fig. 1:**
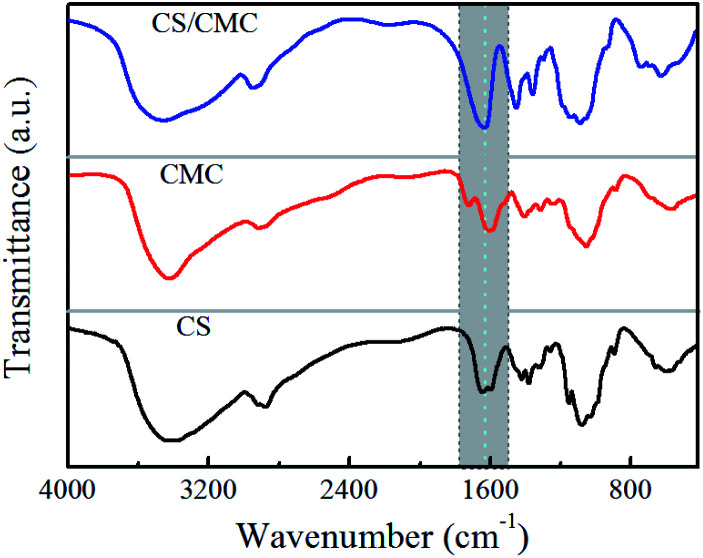
FT-IR spectra of the CS polyelectrolyte coating, CMC polyelectrolyte coating and the CS/CMC multilayer polyelectrolyte coating.

**Scheme 1:**
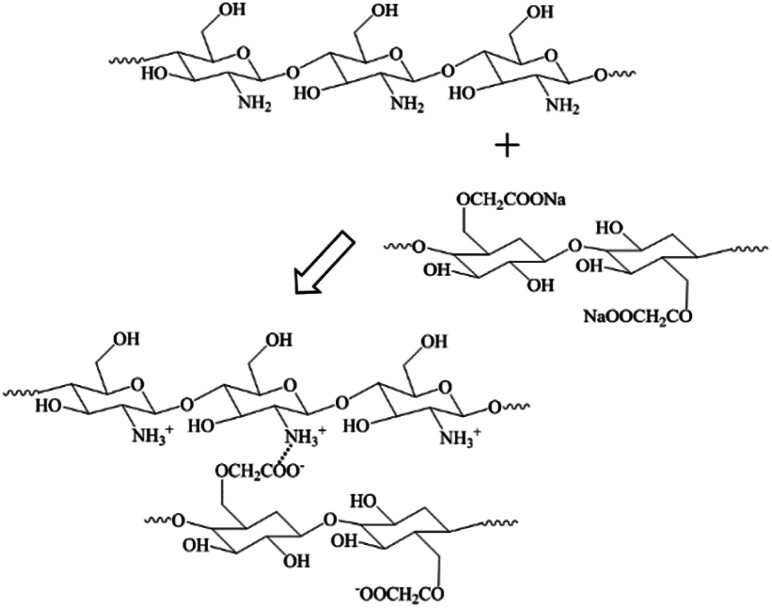
The interaction between CS and CMC.

In this study, the interactions between CS and CMC in the CS/CMC multilayer polyelectrolyte coating were studied *via* infrared spectrometry and the results are shown in Fig. 1. It can be seen from the black line (CS polyelectrolyte coating) that the obvious absorption peaks at 3400 cm^−1^, 1646 cm^−1^ and 1597 cm^−1^, which correspond to N–H, –NHCO– and –NH_2_, are the characteristic absorption peaks of CS. The red line (CMC polyelectrolyte coating) showed obvious absorption peaks at 3400 cm^−1^, 1752 cm^−1^, 1630 cm^−1^ and 1428 cm^−1^, which were the characteristic absorption peaks of CMC, and were all consistent with those reported in Ge's literature.^24^ Comparative observation of the black line and red line with the blue line (CS/CMC multilayer polyelectrolyte coating) revealed that it can be found that the absorption peaks of the amino and carboxyl groups shifted, which indicates that CS and CMC interact through hydrogen bonding.^25^ The hydrogen bonding can undergo a reversible “break-rebuild” process under certain stimulation conditions, which is suitable for the designing of new stimulus-response materials. Recently, a lot of new functional materials with self-healing property were constructed through hydrogen bonding; for example, Sun *et al.* have successfully constructed a coating material with excellent self-healing performance based on the hydrogen bonding between bPEI and PAA.^26–28^ In this study, the possible hydrogen bonding between CS and CMC (Scheme 1) was used to construct a biomedical material with good self-healing properties, extending the life of the material and improving the safety of the material during use.

**Fig. 2:**
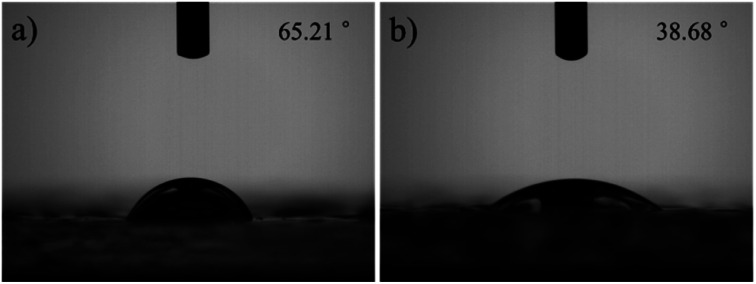
The wettability of the CS/CMC multilayer polyelectrolyte coating fabricated with different pH of CS solution, (a) 4.0, (b) 2.5.

The infiltration of the surface of biomaterials can affect a variety of cell behaviours, such as cell adhesion, cell orientation, cell mobility, cell surface antigen expression, cell surface signal channels, and gene expression. Zhao^29^ found that the better the hydrophobicity of the coating surface, more conducive the attachment and growth of cells on the coating surface, and the better the biocompatibility. In addition, the coatings are repaired under the stimulation of water, so there are certain requirements for the surface wettability of the material. When the wettability of the material is better, the water diffuses faster on the coating surface and is more beneficial for the rapid repair of the material. To achieve this, numerous materials are subjected to surface treatments, such as a thorough plasma treatment, before performing experiments. Therefore, we tested the wettability of the CS/CMC polyelectrolyte multilayer coatings, and the relevant results are shown in Fig. 2. It can be found from Fig. 2(b) that the contact angle of the CS/CMC polyelectrolyte multilayer coating is 38.68° in the chitosan solution with a pH of 2.5, which is very helpful to improve the self-healing performance and the bio-fusion properties of the CS/CMC polyelectrolyte multilayer coating.

This is because chitosan is a polyelectrolyte material and its conformation behaviour is easily affected by H^+^. When the proton intensity is large, the conformation exhibits a more stretched structure, and its wettability tends to be better. When the proton intensity is small, its conformational behaviour changes to the contraction direction, and its wettability becomes worse. When the chitosan solution has a pH of 2.5, its conformational behaviour is completely stretched. Therefore, the as-prepared CS/CMC multilayer polyelectrolyte coating had a low contact angle and good wettability.

**Fig. 3:**
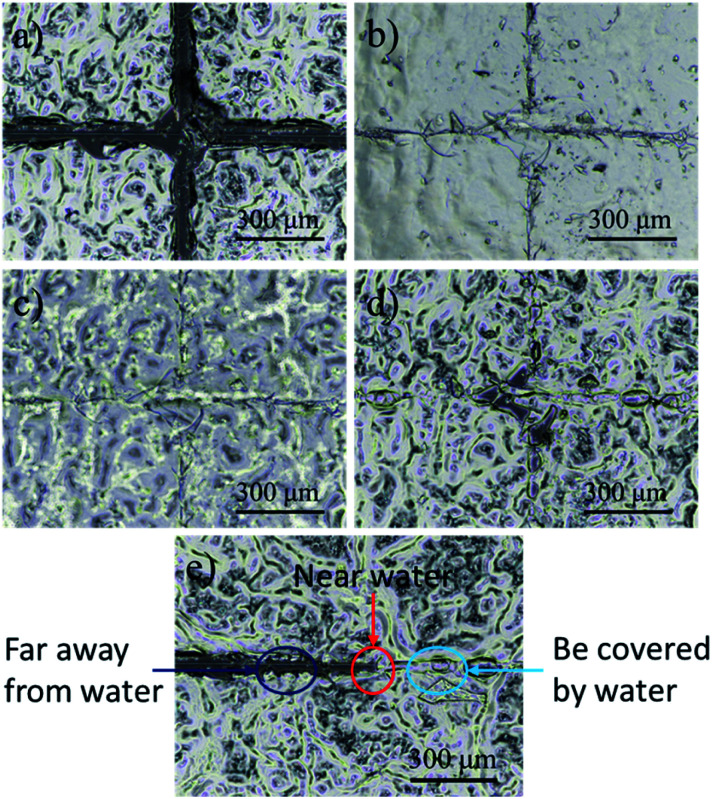
The self-healing process of the CS/CMC polyelectrolyte multilayer coating: (a) damaged, (b and c) self-healing and (d) self- healed. (e) The comparison graph of the repaired and unrepaired parts.

In this experiment, we observed the self-healing process of the CS/CMC polyelectrolyte multilayer coating with the help of a stereomicroscope. The detailed results are shown in the Fig. 3. First, the CS/CMC polyelectrolyte multilayer coating was damaged to a “cross” pattern, which distance was about 30 μm, and penetrated to the substrate (Fig. 3(a)); then, distilled water was gradually dripped on the coating, and the damaged parts gradually absorbed the water, which caused their own volume to expand, and came to contact with each other slowly. When the damaged “cross” pattern was fulfilled by the water, the “cross” pattern gradually disappeared (Fig. 3(b)). After the removal of water, the surface of the CS/CMC polyelectrolyte multilayer coating gradually became dry, and restored its original rough structure. However, the damaged parts of the CS/CMC polyelectrolyte multilayer coating were not reappeared (Fig. 3(c) and (d)), indicating that the CS/CMC polyelectrolyte multilayer coating has a good self-healing property.

In order to further prove that the CS/CMC polyelectrolyte multilayer coating has the self-healing property, we also observed and photographed the comparison graph of the repaired and unrepaired parts. From the Fig. 3(e), we can observe that the damaged parts of the CS/CMC polyelectrolyte multilayer coating covered by water can completely repair itself. Also, the damaged parts of the CS/CMC polyelectrolyte multilayer coating near the water could also absorb some water, and therefore partly repair itself, decreasing its scratch width. While without water stimulation, the damaged parts of the CS/CMC polyelectrolyte multilayer far away from the water did not change, which further demonstrated that the CS/CMC polyelectrolyte multilayer could undergo the self-healing process in the presence of water.

**Fig. 4:**
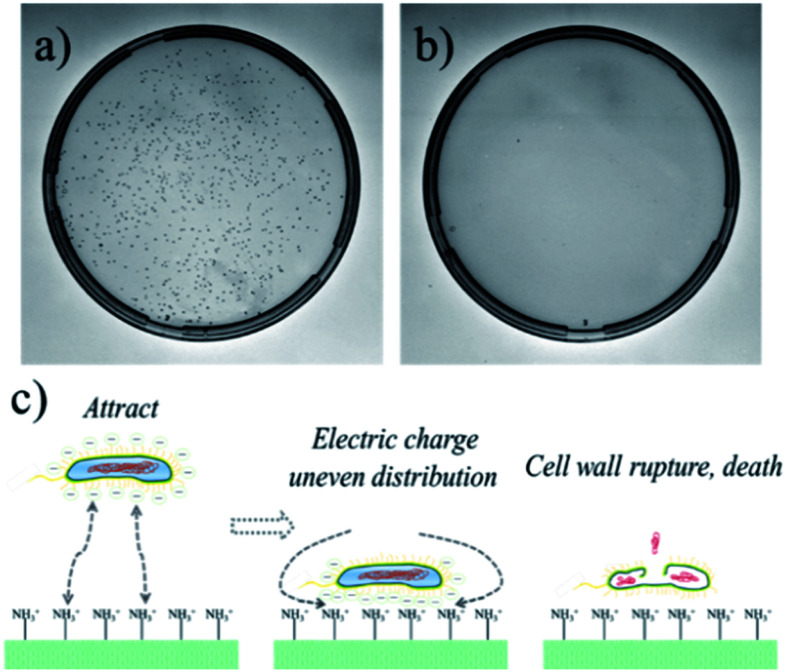
The antibacterial effect of (a) the control sample, (b) the CS/CMC multilayer polyelectrolyte coating, (c) the antibacterial mechanism of the CS/CMC multilayer polyelectrolyte coating.

Medical devices and equipment are in close contact with human tissues during the application process and need to be sterile on the surface.^30^ It is generally required to maintain the aseptic state by aseptic packaging. However, during production, transportation, storage, and particularly during implantation, materials inevitably come into contact with bacteria, which may cause great harm to the human body. Therefore, if the coating can be endowed with antibacterial properties, the application range of the coating material in medical devices and equipment will be further expanded.^31^ Recently, there are numerous reports about the antibacterial properties of the coatings, and the effects are also very promising. However, nanoparticles are usually assembled on the surfaces of the coatings for antibacterial purposes. The introduced nanoparticles have a certain biotoxicity, particularly the Ag nanoparticles, so their further application is limited. In this experiment, the CS and CMC were chosen as assembly motifs to construct CS/CMC antibacterial coating under specific pH conditions, and its antibacterial properties against *E. coli* was studied. The antibacterial results are shown in Fig. 4 and S1.† It can be seen in Fig. 4(a) that the culture medium of the control group is full of *E. coli*, while the culture medium containing the CS/CMC (in a specific amount) sample can only find fewer colonies (Fig. 4(b)). This shows that the CS/CMC polyelectrolyte multilayer coating has a strong antibacterial property. Its antibacterial principle is shown in Fig. 4(c). The assembly element CS we selected contains a large number of amino groups. When *E. coli* is attached to the coating surface, the positive charge of the amino group can adsorb the negative charge on the surface of *E. coli*, disrupting the charge balance on the surface of the bacteria, and cause *E. coli* to lyse and death.

As the CS/CMC polyelectrolyte multilayer coating is designed based on the unique properties of CS, it not only has good antibacterial properties, but also overcomes the antibacterial biological toxicity of traditional Ag nanoparticles. Therefore, it has broad application prospects in the field of biocompatible coatings.

**Fig. 5:**
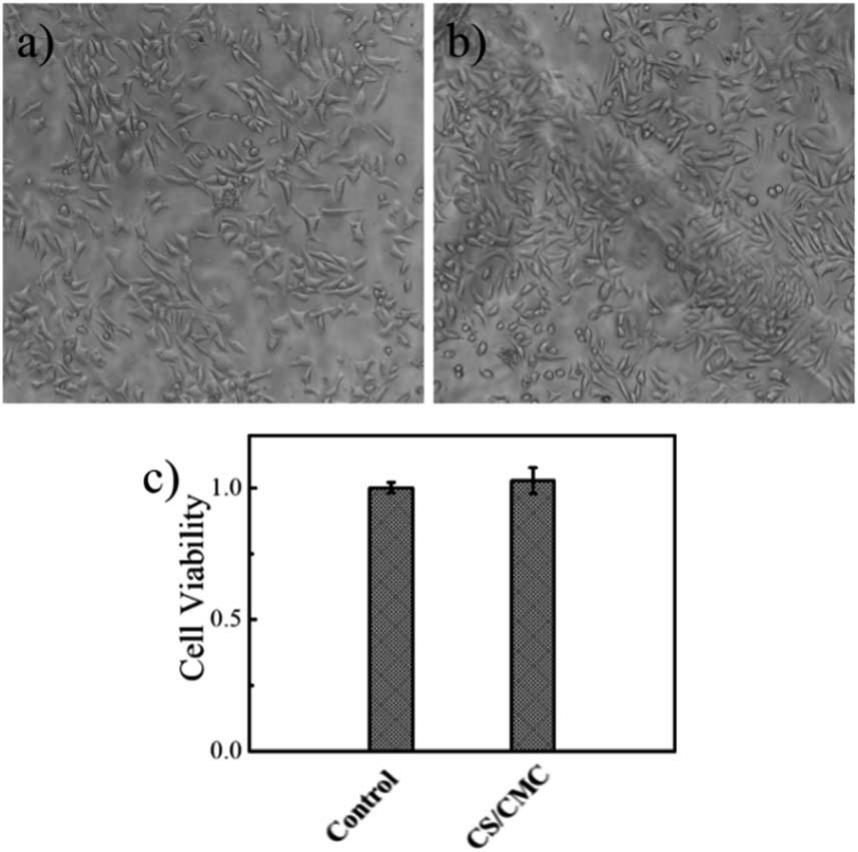
The biocompatibility of (a) the 24-well plate, (b) the CS/CMC multilayer polyelectrolyte coating and (c) cck-8 results.

The surface modification technology is gradually used to improve the antibacterial performance, surface lubrication, and mechanical properties of biomaterials. However, the premise of the surface modification technology is the good biocompatibility of the coating. For this purpose, we investigated the biocompatibility of the CS/CMC polyelectrolyte multilayer coating. The specific experimental procedure was to culture L929 cells on a 24-well plate coated with the CS/CMC self-healing polyelectrolyte coating for 72 h. The growth state of L929 cells was observed and recorded through a microscope. The results are shown in Fig. 5. From Fig. 5(a) and (b). It can be seen that the L929 cells in the blank 24-well plate are long spindle-shaped, uniform in shape, grow close to the 24-well plate, and are densely arranged on the 24-well plate. The L929 cells on the 24-well plate coated with the CS/CMC self-healing polyelectrolyte coating are also spindle-shaped. Not only the cell shape, but also the cell growth state and cell density are similar to the control group, which indicates that the as-prepared CS/CMC self-healing polyelectrolyte coating has a good biocompatibility.

In addition, we further investigated the biocompatibility of the as-prepared CS/CMC self-healing polyelectrolyte coating through CCK-8 experiments. The results are shown in Fig. 5(c). As compared to the control group, the cell viability in the CS/CMC self-healing polyelectrolyte coating was almost the same as the cell viability in the blank 24-well plate, which further verified the CS/CMC self-healing polyelectrolyte coatings have excellent biocompatibility.

Because the CS/CMC polyelectrolyte multilayer coating not only has good self-repairing properties, antibacterial properties but also good biocompatibility, this guarantees for the application of coatings in biological materials.

## 4 Conclusions

Herein, a highly efficient and technically simple approach was developed to facilely fabricate an antibacterial and biocompatible multilayer biomedical material capable of healing damages. The synthetic strategy involves the alternate deposition of CS and CMC *via* the LBL self-assembly technique. As both the CS and CMC are derived from organisms, the composite materials had high biocompatibility. Further, CS and CMC are bonded by hydrogen bonds and electrostatic attraction, and these reversible non-covalent bonds can be broken and recombined under specific stimuli. Moreover, the CS/CMC multilayer polyelectrolyte coating can adsorb the negative charge on the surface of bacteria, disrupting the charge balance on the surface of the bacteria and causing bacteria to lyse and death. These features enable the CS/CMC multilayer polyelectrolyte coating to have extended lifespan and to be highly promising in the application as novel functional biomedical materials.

## Conflicts of interest

There are no conflicts to declare.

## Supplementary Material

RA-010-D0RA04457A-s001

RA-010-D0RA04457A-s002
