# Supplementary material for: An antibacterial and biocompatible multilayer biomedical coating capable of healing damages
Source: RSC Adv. 2020 Aug 28;10(53):32011–5. doi: 10.1039/d0ra04457a (PMC9056529; doi:10.1039/d0ra04457a)
Supplement: RA-010-D0RA04457A-s002 [file RA-010-D0RA04457A-s002.pdf]

Electronic Supplementary Material (ESI) for RSC Advances.
This journal is © The Royal Society of Chemistry 2020

Supporting information

We have compared the different antibacterial properties between the CS/CMC polyelectrolyte multilayer coating which assembled on glass side and the glass slide in the early stage. However, because the CS/CMC polyelectrolyte multilayer coating only produced antibacterial effects on the part be covered by it, and did not produce a significant inhibition zone. Compared with the glass slide, the antibacterial effect of the CS/CMC polyelectrolyte multilayer coating is not obvious, the preliminary experimental results are shown in figure S1. So we chose to grind the CS/CMC polyelectrolyte multilayer coating into a powder and then disperse it in a bacterial culture dish for antibacterial experiments, and achieved a more obvious antibacterial effect.

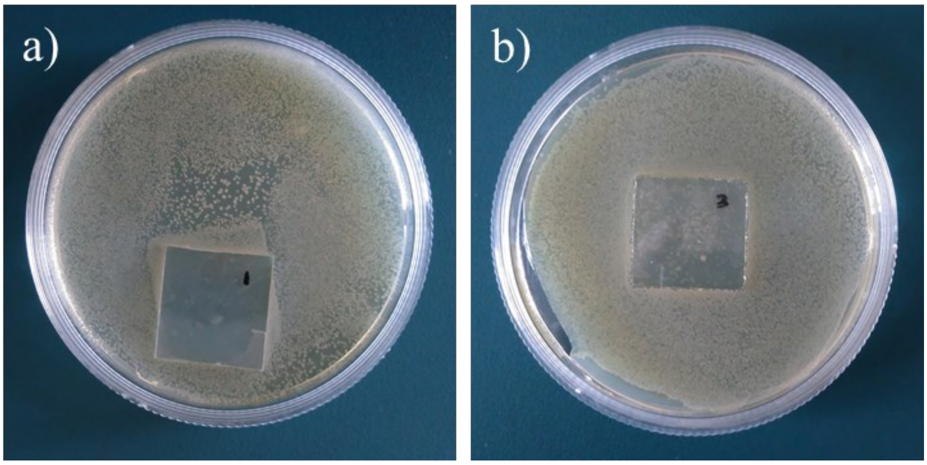

Figure S1. The preliminary antimicrobial test results of (a) CS/CMC polyelectrolyte multilayer coating and (b) glass slide.
